# The Science of Learning and Art of Education in Cardiology Fellowship

**DOI:** 10.14797/mdcvj.1088

**Published:** 2022-06-03

**Authors:** Natalie Stokes, Kathryn Berlacher

**Affiliations:** 1 University of Pittsburgh Medical Center, Pittsburgh, Pennsylvania, US

**Keywords:** medical education, adult learning, cardiology training

## Abstract

The science of learning, bolstered by the foundational principles of adult learning, has evolved to allow for a more sophisticated understanding of how humans acquire knowledge. To optimize learning outcomes, cardiology educators should be familiar with these concepts and apply them routinely when teaching trainees. This paper presents an overview of the neurobiology of learning and adult learning principles and offers examples of ways in which this science can be applied in cardiology fellowships. Both fellows and educators benefit from the science of learning and its artistic application to education.

## Background

Cardiology fellowship remains one of the most competitive matches in the nation, and for good reason. The field of cardiology is constantly evolving with extensive scientific discovery and innovative care delivery, which makes training in this area both exhilarating and challenging. The demands on cardiology trainees and educators necessitate the optimal use of time and resources to achieve target learning outcomes.

The science of learning has grown tremendously in the past few decades. Research demonstrates that learning is an active process that directly changes the architecture of the brain.^1^ New concepts surrounding learning provide an opportunity to optimize the educational processes in cardiology fellowship and enhance durable learning for cardiology fellows. Herein we introduce the basics of the science of learning and adult learning theories and provide creative ways for educators to enrich training in cardiovascular fellowship so that the field and our care of patients continue to improve.

## The science of adult learning

### Neurobiology of Learning

The process of learning involves structural and functional synaptic changes in the central nervous system.^1,2,3^ Multiple distinct components of neurons demonstrate use-dependent plasticity.^4^ Data suggests that specific synaptic and nuclear signaling events facilitate acquisition and retention of information.^5^ Improved efficiency in synaptic transmission has been associated with several mechanisms of knowledge construction, including increased neuronal connectivity, faster conduction, improved blood flow to distinct areas of the brain via angiogenesis, and new and strengthened synaptic connections.^6,7,8,9,10,11,12,13,14^

Synaptic modifications have been directly linked to behavioral learning processes in animal models.^10,11^ Human studies using pathology and functional brain imaging have established that different types of learning stimulate different parts of the brain, and distinct parts of the brain are activated when we attempt to retrieve knowledge.^14,15,16,17,18^ Although a great deal remains to be known about the neurobiology of learning, research has established that the brain has the potential for continual development and undergoes reorganization through the process of learning.^1^

#### Cognitive Load Theory

Adults learn, or acquire actionable knowledge, by processing new information and relating it to previously attained knowledge. Cognitive load theory (CLT) identifies two distinct structures of memory that interact with one another during learning: *working memory*, where we hold and process new information, and *long-term memory*, where we store information we have learned.^19,20^ Working memory has a limited capacity at any given time, while long-term memory has unlimited capacity, which allows us to remember things from the past. The brain learns by moving information from working memory to long-term memory. CLT postulates that this process depends on how information is presented and the complexity of the information itself. Total cognitive load is made up of *intrinsic load*, the content to be learned, and *extrinsic load*, how the content is presented. Working memory is confronted with the total cognitive load; thus, if high effort is required to process the extrinsic load, there are less available resources for processing intrinsic load.^19,20,21,22,23^ In other words, the harder a learner has to work to process *how* information is presented, the less memory the learner has available to process the content itself. Educators can improve learning outcomes by limiting extrinsic load so that learners can convert working memory to long-term memory with less effort.

#### Sensory Processing

Traditionally it was believed that individuals have different learning styles that help them learn the best; for instance, some are visual learners, while others learn best by audio input. Despite being repeatedly discredited, the myth that individuals have distinct learning styles persists in much of medical education.^24^ Yet research demonstrates that adult learners learn best by active involvement with material via multiple sensory pathways.^19,24,25,26^ Imaging studies show that the brain responds to novel information with heightened awareness,^1^ and functional neuronal changes are enhanced when the learner is actively engaged.^27^

Providing access to the same information using multiple sensory pathways, a process known as *dual coding*, enhances learner attention, motivation, and retention.^24,28^ Combining words with visuals, for instance, provides greater impact than use of either modality alone. Likewise, the act of writing new content integrates kinesthetic activity with audiovisual presentation of information and optimizes learner attention through participation.^26,29^

#### Environment and Stress

Memory formation is situational and strongly influenced by the learning environment, including how safe and how stressed the learner feels in a given setting. A sense of safety allows for receptivity to learning; in fact, studies demonstrate that a learner’s motivation and creativity in problem solving increase when they feel safe in the educational environment.^30,31^ However, a safe and optimized learning environment does not mean a stress-free environment. A small amount of stress prompts a neurohormonal cascade of increased dopamine levels with subsequent cortisol release that has been shown to rapidly engage and enhance hippocampal and amygdaloid processing, accounting for enhanced memory construction.^32^ Too much stress, though, results in bad memory formation, likely due to the suppression of prefrontal cortex processing.^33^ The Yerkes-Dodson Law Bell Curve, a century-old model displaying a bell-shaped curve relationship between stress and memory formation, has borne out in both animal and human models.^33,34^

#### Repetition and Retrieval

The term *deliberate practice* refers to effortful practice, beyond merely time and exposure, that is demonstrated to achieve expertise in any given field. It is specific and structured with interval increases in complexity of knowledge and skill formation. Provision of immediate and frequent feedback and opportunities for repeated performance to refine behavior are necessary.^35^ The pacing and spacing of repetition are as important as the practice of repetition itself. Spaced practice has robust support in the literature for strengthening long-term memory formation when compared with compressed learning, showing advantages in basic memory tasks, complex conceptualization, and motor skill acquisition.^36,37,38^ The process of interleaving, or studying related skills or concepts in parallel, has likewise demonstrated to improve learning.^37^ Retrieval practice involves attempting to recreate what was previously learned prior to engaging with the material once again. This skill is shown to strengthen long-term memory formation.^39,40,41^ Optimal deliberate practice engages repeated activation of neuronal pathways with spaced trials, interleaving, and retrieval practice.

#### Reward and Reinforcement

The brain has an intrinsic reward system that reinforces behaviors when activated.^42^ Importantly, the reward of achieving a goal can have the same effect as a more tangible award, such as a prize or money.^43^ The brain is constantly engaged in the process of determining relative value of short-term verses long-term rewards. When an adult can see the benefits of learning within the context of larger goals, they are more engaged in the process.^1^ As noted, feedback is an essential part of deliberate practice and can be used to optimize the intrinsic reward system and reinforce positive behaviors.^44^

### Adult Learning Principles

**Figure 1:**
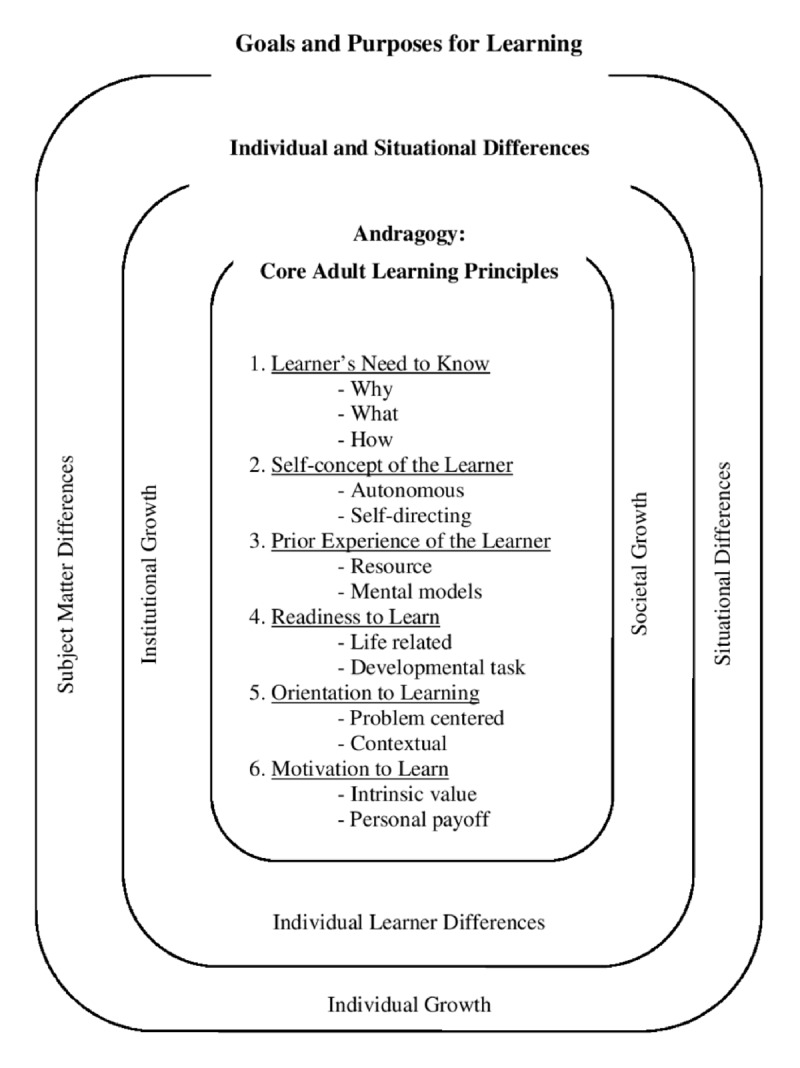
Andragogy in practice model. Reproduced with permission from Knowles et al.^45^

The neurobiology of learning coincides with the development of adult learning principles. These principles translate science into action by providing a foundation for effective teaching. The term “andragogy” was first coined by Malcolm Knowles in the 1970s to differentiate adult learning from “pedagogy” or childhood learning.^45^ Knowles outlined six key learning principles that serve as the foundation of adult learning (***Figure 1***). These principles establish that experience provides the basis for adult learning; adults need to be involved in planning and evaluating their own instruction and are most interested in learning subjects that have immediate relevance to them.^46^

**Figure 2:**
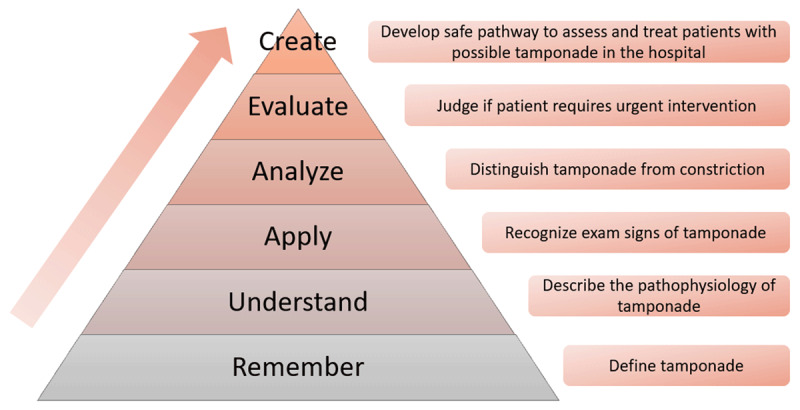
Revised Bloom’s taxonomy with cardiology specific example. Adapted with permission from Krathwohl D.^48^

Benjamin Bloom, in collaboration with other educational scholars, established a framework for categorizing educational goals. Known as Bloom’s taxonomy, it consists of categories of successive skills that make up a continuum of cognitive learning (***Figure 2***).^47^ Recent adaptations include a change in terminology from noun to verb forms and an expanded 2-dimensional framework.^48^ Bloom’s taxonomy is a useful tool in designing and assessing educational curricula.

**Figure 3:**
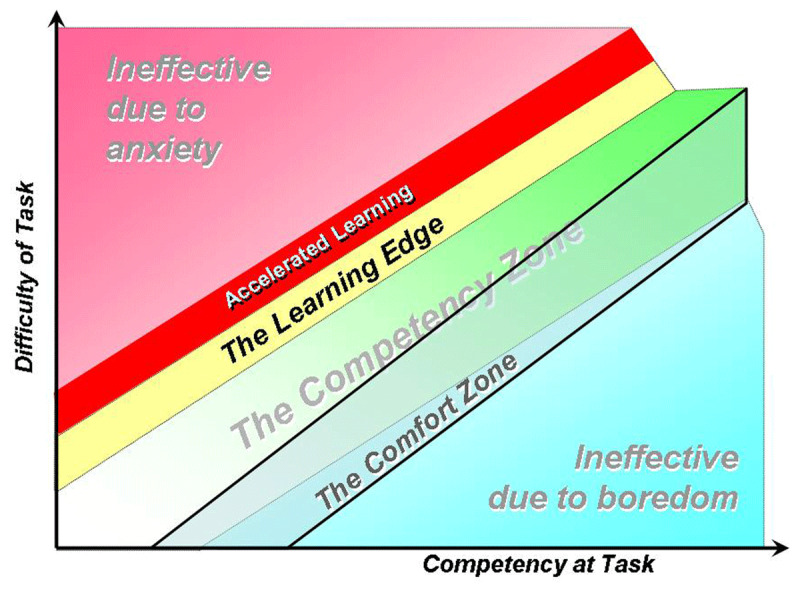
The Comfort Zone and the Learning Edge. Reproduced with permission from Armour.^49^

While not established as a traditional adult learning principle, the concept of the “learning edge” is an optimal adaptation of the aforementioned Yerkes-Dodson Law in practice. Given the unique relationship between stress and knowledge construction, this framework operationalizes the ideal zone of anxiety for knowledge formation. By plotting difficulty of a task versus capability, a competency zone evolves (***Figure 3***). Accelerated learning can occur in this zone, provided the learner is not overly stressed. The learning edge is the point at which a learner is mentally stimulated and challenged, outside of their comfort zone, but still within an environment where they feel unthreatened.^49^

## The art of education

With a more sophisticated understanding of learning, cardiology educators should feel prepared to operationalize these concepts in their day-to-day practice. This science and the principles of adult learning should guide cardiology education across all learners, at all levels, for all rotations in all curricula. In this section, we discuss the art of education, which in essence is the creative application of the science of learning.

Prior to this application, certain fundamentals must be established for fellows to flourish and for education to succeed. First, educators must create a supportive learning environment where fellows can safely address their vulnerabilities and shortcomings and engage in active problem solving. Second, educators should be transparent about the science of learning. Incorporating metacognition—teaching fellows how and why they learn—early in training urges them to capitalize on all learning opportunities and, as outlined below, enhance their independent learning. If fellows know why certain methods are used, they are more likely to participate in the process. Finally, educators must embrace the concept that their role is more closely aligned with that of a facilitator than with owner or distributor of knowledge. Below we provide examples of where and how educators can artistically apply the science of learning in a variety of settings throughout fellowship training.

### Conference Settings

Larger conferences—including grand rounds, journal clubs, and noon conferences—are a mainstay of fellowship education. To optimize these sessions for learning, educators can take some relatively simple steps. First, to help fellows manage intrinsic load, educators should embrace a “content diet.” Key learning objectives should be limited and clearly stated from the start and then repeated at the end of each conference. While it is tempting to be comprehensive about topics when leading a conference, CLT teaches us that providing more material can often be detrimental to true learning given the limited capacity of our working memory. Content that does not directly support stated objectives can be provided as reference material for fellows to review at other times.

Incorporating ways for fellows to be interactive is essential for them to remain attentive and engaged during conferences. While passive didactic lectures increase short-term retention, they are not associated with long-term learning since adults learn better by doing, as noted by Knowles. One simple method of conference interaction proposed by Doug Lemov is “Think-Pair-Share.”^50^ In this approach, fellows are asked to think about a prompt or question. Then they pair with a co-fellow to share their thoughts before reconvening with the entire group. Asking fellows to discuss their thoughts encourages greater participation, builds confidence and creativity in problem solving, and leads to deeper understanding.

***Educator tips*:**

1. ***Less is more*.**
2. ***Interaction increases learning***.

### Clinical Rotations

**Figure 4:**
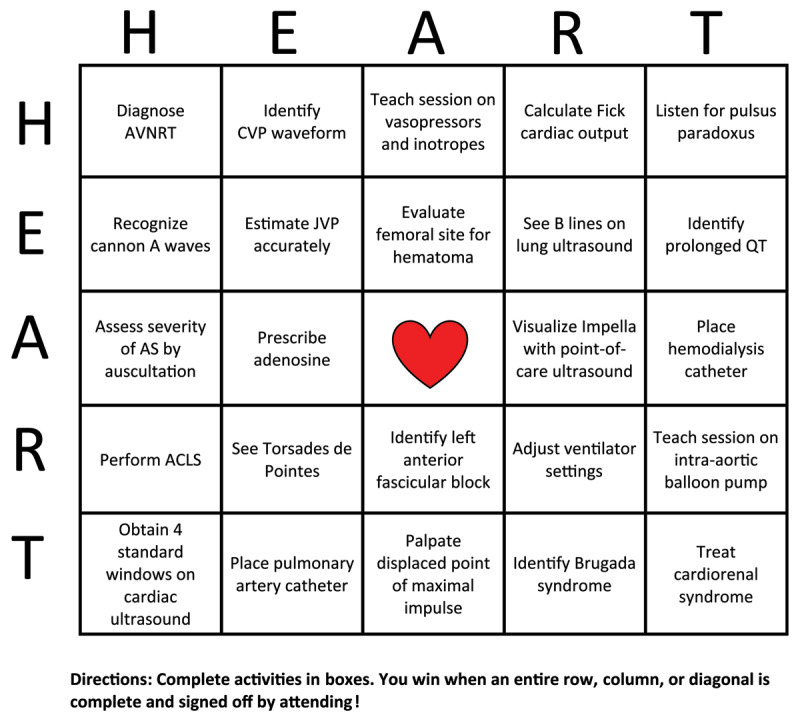
Example of gamification: HEART Bingo for the cardiac critical care unit. AVNRT: atrioventricular nodal reentry tachycardia; CVP: central venous pressure; JVP: jugular venous pressure; AS: aortic stenosis; ACLS: advanced cardiac life support

Much of learning in cardiology fellowship happens spontaneously, such as on rounds, at the bedside, in the echo lab, and alongside the many day-to-day activities that take place throughout training. One way of augmenting learning in these moments is to engage fellows in simple gamification. Gamification applies the standard elements of game playing (ie, badges, levels, leaderboards) to learning and training. While adding a little fun to the workday, gamification also creates a safe learning environment by making failure less risky for fellows. Educators can identify gaps in knowledge without the fellow feeling uncomfortable. Additionally, with competition at play, there is release of dopamine that increases knowledge retention. For example, on the cardiology consult rotation, educators can start the day by asking every member of the team to state one cardiac-related point they learned in the past 24 hours. An appointed medical student can keep track of all learning points throughout the week. At the end of the week, the team can review those points and vote on three favorites. The team members responsible for those points receive bragging rights…and possibly also coffee from the attending that afternoon! As noted, both spaced retrieval (ie, recalling a learning point from the day prior) and repetition with spaced intervals (ie, reviewing points at the end of the week) are important for long-term memory formation.^36,37,38,39,40,41^ Alternatively, an educator can create a quick game of “Heart Bingo” at the start of the week, filling each box with a cardiac-related activity. Members of the team work to complete these activities throughout the week, and the first person to complete an entire row, column, or diagonal wins (***Figure 4***).

***Educator tip: Play more, learn more***.

### Acquiring New Skills

Almost everyone in medicine is familiar with the old motto “see one, do one, teach one.” Yet every cardiologist who has been in a complex percutaneous intervention knows that teaching such procedures is not that simple. A new six-step framework of training procedural competency has been developed by Taylor Sawyer and colleagues using advancements in the science of learning: learn, see, practice, prove, do, and maintain.^51^ The first two steps provide fundamental knowledge and observation of the skill prior to introduction of psychomotor acquisition. Importantly, initial practice for skill development is often performed in a simulated setting where repetitive practice is allowed before fellows prove they are ready to perform the behavior in a clinical setting. All patient interactions must be done under direct supervision with support and guidance as fellows progress to independent practice. Maintenance of skills is achieved by continuous clinical practice and, if necessary, supplementation with simulation-based training.

Feedback is an essential part of skill acquisition for a cardiology trainee. Recently called into question is a commonly taught method of feedback, the “sandwich approach,” wherein the educator provides one constructive or negative piece of feedback sandwiched between two positive comments. This ineffective means of giving feedback, as discussed in many business articles and blogs, undermines both the content of the feedback and the relationship educators have with fellows.^52^ A better feedback framework is Ask, Tell, Ask.^53^ First, the fellow is asked to name one thing they did well and one thing they could do better. Second, the fellow is told how they did, using specific behavior-based examples. Finally, the fellow is asked to postulate specific ways they can improve, making an action plan together with the educator.

***Educator tip: Give feedback on observed skills and then repeat, repeat, repeat*.**

### Daily Fellow Routines

To attain competency in cardiology and to continue that competency for the duration of a career, fellows must practice routine and effective skills for independent learning during training. Passive reading and reviewing content for hours rarely translates to significant retention. Fellows should be encouraged to find ways to make content and concepts stick. Thus, educators should deliberately discuss and model practices that lead to effective learning. As noted, the simple kinesthetic activity of using a pen and paper to write down learning points activates multiple sensory pathways, allowing for increased likelihood of retention.^26,29^ Educators should write things down when encountering new content and ask fellows to do the same.

If facilitating board preparation sessions, educators should integrate multiple areas of cardiology rather than covering a single topic. This incorporates the concept of interleaving. When planning noon conferences and grand rounds schedules, revisit topics frequently to allow for spaced repetition. Before revisiting topics, fellows should be encouraged to recall what has been previously taught on the topic, fostering retrieval practice. This can be done both in group sessions and at home when the fellow is independently studying. Finally, fellows should be inspired to climb Bloom’s taxonomy pyramid regarding the content being learned (***Figure 2***). Evaluation and creation of cardiology concepts are the primary goals for fellows during training.

***Educator tip: Form habits that make concepts stick***.

## Conclusion

As the fields of cardiology and education science evolve, informed and inventive educators need to be at the helm of cardiology training. Advances in the science of learning provide a wealth of opportunity to enhance education during cardiology fellowship. When education is done well, the acquisition of cardiac knowledge is more consistent and efficient, benefitting not only fellows but also our patients.

## Key Points

- Advances in understanding the neurobiology of learning provide foundational structure for adult learning theories.
- The principles of adult learning theory should guide education of all learners in all contexts during cardiovascular fellowship.
- The art of education is in the application of the science of adult learning.
- A supportive learning environment and metacognition are required when optimizing learning outcomes.
- Multiple opportunities exist to optimize education in cardiology fellowship, including during conferences, on rounds, when teaching new skills, and when structuring independent learning.

## References

[1] Friedlander MJ, Andrews L, Armstrong EG, et al. What can medical education learn from the neurobiology of learning? Acad Med. 2011 Apr;86(4):415–20. doi: 10.1097/ACM.0b013e31820dc197 21346504

[2] Bourne JN, Harris KM. Balancing structure and function at hippocampal dendritic spines. Annu Rev Neurosci. 2008;31:47–67. doi: 10.1146/annurev.neuro.31.060407.125646 18284372 PMC2561948

[3] De Roo M, Klauser P, Garcia PM, Poglia L, Muller D. Spine dynamics and synapse remodeling during LTP and memory processes. Prog Brain Res. 2008;169:199–207. doi: 10.1016/S0079-6123(07)00011-8 18394475

[4] Kopec CD, Real E, Kessels HW, Malinow R. GluR1 links structural and functional plasticity at excitatory synapses. J Neurosci. 2007 Dec 12;27(50):13706–18. doi: 10.1523/JNEUROSCI.3503-07.2007 18077682 PMC6673607

[5] Lee YS, Silva AJ. The molecular and cellular biology of enhanced cognition. Nat Rev Neurosci. 2009 Feb;10(2):126–40. doi: 10.1038/nrn2572 19153576 PMC2664745

[6] Newpher TM, Ehlers MD. Spine microdomains for postsynaptic signaling and plasticity. Trends Cell Biol. 2009 May;19(5):218–27. doi: 10.1016/j.tcb.2009.02.004 19328694

[7] Eisch AJ, Cameron HA, Encinas JM, Meltzer LA, Ming GL, Overstreet-Wadiche LS. Adult neurogenesis, mental health, and mental illness: hope or hype? J Neurosci. 2008 Nov 12;28(46):11785–91. doi: 10.1523/JNEUROSCI.3798-08.2008 19005040 PMC2793333

[8] Li G, Pleasure SJ. Ongoing interplay between the neural network and neurogenesis in the adult hippocampus. Curr Opin Neurobiol. 2010 Feb;20(1):126–33. doi: 10.1016/j.conb.2009.12.008 20079627 PMC2837845

[9] Zhao C, Deng W, Gage FH. Mechanisms and functional implications of adult neurogenesis. Cell. 2008 Feb 22;132(4):645–60. doi: 10.1016/j.cell.2008.01.033 18295581

[10] Kim SJ, Linden DJ. Ubiquitous plasticity and memory storage. Neuron. 2007 Nov 21;56(4):582–92. doi: 10.1016/j.neuron.2007.10.030 18031678

[11] Lynch G, Rex CS, Gall CM. LTP consolidation: substrates, explanatory power, and functional significance. Neuropharmacology. 2007 Jan;52(1):12–23. doi: 10.1016/j.neuropharm.2006.07.027 16949110

[12] Pagani MR, Oishi K, Gelb BD, Zhong Y. The phosphatase SHP2 regulates the spacing effect for long-term memory induction. Cell. 2009 Oct 2;139(1):186–98. doi: 10.1016/j.cell.2009.08.033 19804763 PMC2770243

[13] Yang G, Pan F, Gan WB. Stably maintained dendritic spines are associated with lifelong memories. Nature. 2009 Dec 17;462(7275):920–4. doi: 10.1038/nature08577 19946265 PMC4724802

[14] Lynch G, Rex CS, Gall CM. LTP consolidation: substrates, explanatory power, and functional significance. Neuropharmacology. 2007 Jan;52(1):12–23. doi: 10.1016/j.neuropharm.2006.07.027 16949110

[15] Snyder JS, Glover LR, Sanzone KM, Kamhi JF, Cameron HA. The effects of exercise and stress on the survival and maturation of adult-generated granule cells. Hippocampus. 2009 Oct;19(10):898–906. doi: 10.1002/hipo.20552 19156854 PMC2755652

[16] Konur S, Yuste R. Imaging the motility of dendritic protrusions and axon terminals: roles in axon sampling and synaptic competition. Mol Cell Neurosci. 2004 Dec;27(4):427–40. doi: 10.1016/j.mcn.2004.07.005 15555921

[17] Gallo DA, McDonough IM, Scimeca J. Dissociating source memory decisions in the prefrontal cortex: fMRI of diagnostic and disqualifying monitoring. J Cogn Neurosci. 2010 May;22(5):955–69. doi: 10.1162/jocn.2009.21263 19413478

[18] Crescentini C, Shallice T, Del Missier F, Macaluso E. Neural correlates of episodic retrieval: an fMRI study of the part-list cueing effect. Neuroimage. 2010 Apr 1;50(2):678–92. doi: 10.1016/j.neuroimage.2009.12.114 20060480

[19] Willingham DT. Why Don’t Students Like School? A Cognitive Scientist Answers Questions About How the Mind Works and What It Means for the Classroom. San Francisco, CA: Jossey-Bass; 2010. 240 p.

[20] Sweller J, Van Merrienboer JJ, Paas FGWC. Cognitive architecture and instructional design. Educ Psychol Rev. 1998 Sep;10:251–296.

[21] Van Merriënboer JJ, Sweller J. Cognitive load theory in health professional education: design principles and strategies. Med Edu. 2010 Jan;44(1):85–93. 10.1111/j.1365-2923.2009.03498.x 20078759

[22] Gooding HC, Mann K, Armstrong E. Twelve tips for applying the science of learning to health professions education. Med Teach. 2017 Jan;39(1):26–31. doi: 10.1080/0142159X.2016.1231913 27665669

[23] Young JQ, Van Merrienboer J, Durning S, Ten Cate O. Cognitive load theory: implications for medical education: AMEE Guide No. 86. Med Teach. 2014 May;36(5):371–84. doi: 10.3109/0142159X.2014.889290 24593808

[24] Dembo M, Howard K. Advice about the Use of Learning Styles: A Major Myth in Education. J Coll Read. 2007 Jul;37(2):101–109. doi: 10.1080/10790195.2007.10850200

[25] Cabeza R, Ciaramelli E, Olson IR, Moscovitch M. The parietal cortex and episodic memory: An attentional account. Nat Rev Neurosci. 2008 Aug;9(8):613–25. doi: 10.1038/nrn2459 18641668 PMC2692883

[26] Sousa DA. Mind, brain and education: Neuroscience implications for the classroom. Bloomington, IN: Solution Tree Press; 2010. 312 p.

[27] Baumann O, Chan E, Mattingley JB. Dissociable neural circuits for encoding and retrieval of object locations during active navigation in humans. Neuroimage. 2010 Feb 1;49(3):2816–25. doi: 10.1016/j.neuroimage.2009.10.021 19837178

[28] Mayer RE, Anderson RB. The instructive animation: helping students build connections between words and pictures in multimedia learning. J Educ Psychol. 1992;84(4):444–452. doi: 10.1037/0022-0663.84.4.444

[29] Carey B. How We Learn: The Surprising Truth about When, Where and Why it Happens. New York, NY: Random House Trade Paperbacks; 2014. 272 p.

[30] Ashaeur SA, Macan T. How can leaders foster team learning? Effects of leader-assigned mastery and performance goals and psychological safety. J Psychol. Nov-Dec 2013;147(6):541–61. doi: 10.1080/00223980.2012.719940 24199511

[31] Hesketh EA, Bagnall G, Buckley EG, et al. A framework for developing excellence as a clinical educator. Med Educ. 2001 Jun;35(6):555–64. doi: 10.1046/j.1365-2923.2001.00920.x 11380858

[32] Cazakoff BN, Howland JG. Acute stress disrupts paired pulse facilitation and long-term potentiation in rat dorsal hippocampus through activation of glucocorticoid receptors. Hippocampus. 2010 Dec;20(12):1327–31. doi: 10.1002/hipo.20738 20043285

[33] Diamond DM, Campbell AM, Park CR, Halonen J, Zoladz PR. The temporal dynamics model of emotional memory processing: a synthesis on the neurobiological basis of stress-induced amnesia, flashbulb and traumatic memories, and the Yerkes-Dodson law. Neural Plast. 2007;2007:60803. doi: 10.1155/2007/60803 17641736 PMC1906714

[34] Yerkes RM, Dodson JD. The relation of strength of stimulus to rapidity of habit-formation. Punishment: Issues and experiments. 1908 Nov;18(5):459–482. doi: 10.1002/cne.920180503

[35] Ericsson KA. Acquisition and maintenance of medical expertise: a perspective from the expert-performance approach with deliberate practice. Acad Med. 2015 Nov;90(11):1471–86. doi: 10.1097/ACM.0000000000000939 26375267

[36] Benjamin AS, Tullis J. What makes distributed practice effective? Cogn Psychol. 2010 Nov;61(3):228–47. doi: 10.1016/j.cogpsych.2010.05.004 20580350 PMC2930147

[37] Dunlosky J, Rawson KA, Marsh EJ, Nathan MJ, Willingham DT. Improving students’ learning with effective learning techniques: promising directions from cognitive and educational psychology. Psychol Sci Public Interest. 2013 Jan;14(1):4–58. doi: 10.1177/1529100612453266 26173288

[38] Cepeda NJ, Vul E, Rohrer D, Wixted JT, Pashler H. Spacing effects in learning: a temporal ridgeline of optimal retention. Psychol Sci. 2008 Nov;19(11):1095–102. doi: 10.1111/j.1467-9280.2008.02209.x 19076480

[39] Roediger HL 3rd, Putnam AL, Smith MA. Ten benefits of testing and their applications to educational practice. In: Mestre JP, Ross BH, editors. The psychology of learning and motivation: Cognition in education. Cambridge, MA: Academic Press; pp. 1–36. doi: 10.1016/B978-0-12-387691-1.00001-6

[40] Soderstrom NC, Kerr TK, Bjork RA. The Critical Importance of Retrieval--and Spacing--for Learning. Psychol Sci. 2016 Feb;27(2):223–30. doi: 10.1177/0956797615617778 26674128

[41] Kornell N, Hays MJ, Bjork RA. Unsuccessful retrieval attempts enhance subsequent learning. J Exp Psychol Learn Mem Cogn. 2009 Jul;35(4):989–98. doi: 10.1037/a0015729 19586265

[42] Fields HL, Hjelmstad GO, Margolis EB, Nicola SM. Ventral tegmental area neurons in learned appetitive behavior and positive reinforcement. Annu Rev Neurosci. 2007;30:289–316. doi: 10.1146/annurev.neuro.30.051606.094341 17376009

[43] Izuma K, Saito DN, Sadato N. Processing of social and monetary rewards in the human striatum. Neuron. 2008 Apr 24;58(2):284–94. doi: 10.1016/j.neuron.2008.03.020 18439412

[44] ERIC [Internet]. Washington, DC: US Department of Education; c2022. Beesley AD, Apthorp HS. Classroom Instruction That Works. Mid-continent Research for Education and Learning; 2010 Nov 30 [cited 2022 Apr 13]. Available from: https://eric.ed.gov/?id=ED543521

[45] Knowles M, Holton EI, Swanson R. The adult learner: the definitive classic in adult education and human resource development. Burlington, MA: Elsevier; 2005. 392 p.

[46] Taylor DCM, Hamdy H. Adult learning theories: implications for learning and teaching in medical education: AMEE Guide No. 83. Med Teach. 2013 Nov;35(11):e1561–72. doi: 10.3109/0142159X.2013.828153 24004029

[47] Bloom BS, Kratwohl DR, Masia BB. Taxonomy of educational objectives: the classification of educational goals. New York, NY: Longmans Publishing; 1984.

[48] Krathwohl D. A Revision of Bloom’s Taxonomy: An Overview. Theory Into Practice. 2002;41(4):212–218. doi: 10.1207/s15430421tip4104_2

[49] Armour P. The Learning Edge. Communications of the ACM. 2006 Jun;49(6):19–22. Available from: https://web.eecs.umich.edu/~imarkov/Learn_SW.pdf

[50] Lemov D. Teach Like a Champion: 49 Techniques that Put Students on the Path to College (K-12). Hoboken, NJ: Jossey-Bass; 2010. 352 p.

[51] Sawyer T, White M, Zaveri P, et al. Learn, see, practice, prove, do, maintain: an evidence-based pedagogical framework for procedural skill training in medicine. Acad Med. 2015 Aug;90(8):1025–33. doi: 10.1097/ACM.0000000000000734 25881645

[52] HBR.org [Internet]. Cambridge, MA: Harvard Business School Publishing; c2022. Schwartz R. The “Sandwich Approach” Undermines Your Feedback; 2013 Apr 19 [cited 2022 Apr 3]. Available from: https://hbr.org/2013/04/the-sandwich-approach-undermin

[53] Columbia.edu [Internet]. New York, NY: Columbia University; c2022. Konopasek L, Mutnick A, Encandela J, Pica G. Beyond the Feedback Sandwich: Fun Tools for Improving Feedback Skills. Available from: https://resteach.ccnmtl.columbia.edu/mod4/Mod4_Feedback.ppt

